# The efficacy of early progressive resistance exercise in the postoperative management of pancreaticoduodenectomy for pancreatic cancer: a randomized controlled trial

**DOI:** 10.3389/fsurg.2025.1609788

**Published:** 2025-08-01

**Authors:** Xuexue Liu, Neng Shi, Rui Li, Yuan Song

**Affiliations:** Department of Pancreas Center, Nanjing BenQ Medical Center, The Affiliated BenQ Hospital of Nanjing Medical University, Nanjing, Jiangsu, China

**Keywords:** progressive resistance exercise, cancer, postoperative, clinical, nursing, care

## Abstract

**Background:**

Enhanced Recovery After Surgery (ERAS) is of significant importance to the prognosis of patients with pancreatic cancer who have undergone pancreaticoduodenectomy. This study aims to analyze the efficacy of early progressive resistance exercise in the postoperative management of pancreaticoduodenectomy for pancreatic cancer, thereby providing evidence-based support for clinical treatment and nursing.

**Methods:**

This study enrolled patients who underwent pancreaticoduodenectomy for pancreatic cancer at our hospital from January 2023 to December 2024. Participants were randomized into two groups using a random number table: the progressive resistance exercise group and the control group. The control group received standard care, while the progressive resistance exercise group underwent the progressive resistance exercise protocol in addition to standard care.

**Results:**

A total of 80 patients who underwent pancreaticoduodenectomy for pancreatic cancer were included in the study, with 40 patients assigned to each group. Progressive resistance exercise significantly reduced the time to first water intake, time to first food intake, time to first ambulation, and duration of hospital stay (all *p* < 0.05). Post-intervention, the EORTC QLQ-C30 scores increased in both groups, with the progressive resistance exercise group achieving significantly higher scores (*p* < 0.05). Concurrently, the VAS and PSQI scores decreased in both groups, with the progressive resistance exercise group exhibiting significantly lower scores (*p* < 0.05). Scores for emotional state, physical comfort, psychological support, self-care ability, and pain all improved post-intervention, with the progressive resistance exercise group showing significantly higher scores (*p* < 0.05). Additionally, the incidence of urinary retention was significantly lower in the progressive resistance exercise group (*p* = 0.011).

**Conclusion:**

Progressive resistance exercise has been demonstrated to effectively promote functional recovery and overall rehabilitation in patients with pancreatic cancer following pancreaticoduodenectomy. Given its significant benefits, the integration of progressive resistance exercise into clinical practice and nursing protocols is recommended.

## 1 Introduction

Pancreatic cancer is one of the most aggressive malignancies in the digestive system, with a persistently rising incidence rate globally (1). Annually, there are approximately 496,000 new cases of pancreatic cancer worldwide, resulting in around 466,000 deaths (2). The case-fatality rate, which is the ratio of mortality to incidence, is as high as 0.94, making it the cancer with the worst prognosis among all malignancies (3). Owing to the deep anatomical location of the pancreas and the lack of specific clinical manifestations in the early stages, about 80% of patients are diagnosed at a locally advanced or metastatic stage, with a 5-year survival rate of only 7%–9% (4). Statistics from the American Cancer Society show that the incidence of pancreatic cancer is increasing at a rate of approximately 0.5%−1% per year, and it is projected to become the second leading cause of cancer-related death by 2030 (5). This grim epidemiological situation not only imposes a heavy disease burden but also poses significant challenges to the allocation of medical system resources (6).

Pancreaticoduodenectomy (PD), as the only potentially curative treatment for resectable pancreatic head cancer, can significantly reduce tumor burden. However, it is a complex and invasive procedure, with a postoperative complication rate as high as 30% (7). Data from the International Study Group of Pancreatic Surgery (ISGPS) indicate that the incidence of clinically relevant pancreatic fistula (CR-POPF) is about 15%–20%, and the incidence of delayed gastric emptying (DGE) is approximately 20%–30%, both of which severely affect postoperative recovery (8). More importantly, surgical stress can lead to persistent immune suppression, characterized by an imbalance in the CD4+/CD8+ T-cell ratio and reduced NK cell activity, a state that can last for more than 4 weeks after surgery (9). Traditional passive rehabilitation models often fail to effectively improve this condition (10). In contrast, early progressive resistance exercise based on the Enhanced Recovery After Surgery (ERAS) concept, which regulates the IL-6/STAT3 signaling pathway and the muscle-immune axis, has demonstrated unique advantages in postoperative recovery of colorectal cancer and other conditions (11). However, its application effect in pancreatic cancer patients after PD remains to be supported by high-level evidence-based medical evidence. Therefore, this study aims to enroll a number of patients after PD to systematically evaluate the clinical value of the progressive resistance exercise protocol, with the expectation of providing a new intervention strategy for optimizing perioperative management of pancreatic cancer.

## 2 Methods

### 2.1 Ethical consideration

This study was designed as a randomized controlled trial. The protocol was reviewed and approved by the Medical Ethics Committee of The Affiliated BenQ Hospital of Nanjing Medical University (approval number: 2025-KL007). Written informed consent was obtained from all participants, and the data collected were used exclusively for the purposes of this study.

### 2.2 Population

Patients who had undergone pancreaticoduodenectomy for pancreatic cancer at our hospital between January 2023 and December 2024 were recruited for this study. The inclusion criteria for participants in this study were as follows: patients who met the diagnostic criteria for pancreatic cancer as specified in the guideline (12); those who were eligible for and had successfully undergone pancreaticoduodenectomy; individuals with normal mental, neurological, language, and auditory functions and the ability to comply with the interventions in the study; patients aged over 18 and up to 70 years; those with normal coagulation function; patients with a predicted survival time of at least 3 months; individuals with stable vital signs; those with normal function of other vital organs; and patients who had been fully informed about the study and had given their consent to participate.

The exclusion criteria were as follows: patients with a history of limb dysfunction; those with combined immune dysfunction or severe infection; patients with recurrent pancreatic cancer; individuals with other malignancies; and patients who were unwilling to participate in this study.

### 2.3 Interventions

Patients who met the relevant criteria and were enrolled in this study were assigned to either the Resistance exercise group or the Control group according to a computer-generated random sequence. The Control group received standard care. After undergoing pancreaticoduodenectomy, the vital signs of patients in the Control group were monitored using an 866Q64 multiparameter monitor (manufactured by Mindray Bio-Medical Electronics Co., Ltd., Shenzhen, China). Conventional analgesic treatment was administered via intravenous infusion. The fixation of the drainage tube was closely observed to ensure it remained patent. In accordance with medical orders, patients were assisted to turn over and maintained in a semi-recumbent position 6 h after regaining consciousness from anesthesia. Patients were required to fast postoperatively and were provided with nutritional support through intravenous infusion. They were allowed to consume small amounts of liquid food after the recovery of gastrointestinal motility. Based on the patients' tolerance, they were assisted to get out of bed and move around 3–4 days after surgery to prevent the formation of thrombotic diseases. The insurance payment was covered through standard hospital procedures and patient insurance plans.

In our institution, the standardized postoperative management protocol for patients undergoing pancreaticoduodenectomy is designed to optimize recovery and minimize complications. This protocol includes early mobilization to enhance physical recovery, multimodal analgesia to effectively manage pain, and a gradual dietary progression to support gastrointestinal function. Additionally, patients are closely monitored for potential complications, such as pancreatic fistula, infection, and delayed gastric emptying.

All patients included in this study underwent a consistent surgical procedure, specifically an open pancreaticoduodenectomy. The majority of patients received a classical Whipple procedure, while a smaller subset underwent pylorus-preserving resection, based on the specific tumor location and the surgeon's clinical judgment. A midline laparotomy was used uniformly for surgical access. In terms of perioperative management, all patients received general anesthesia with endotracheal intubation, intraoperative fluid management guided by hemodynamic monitoring, and postoperative analgesia that combined opioids with non-opioid analgesics.

**Figure 1:**
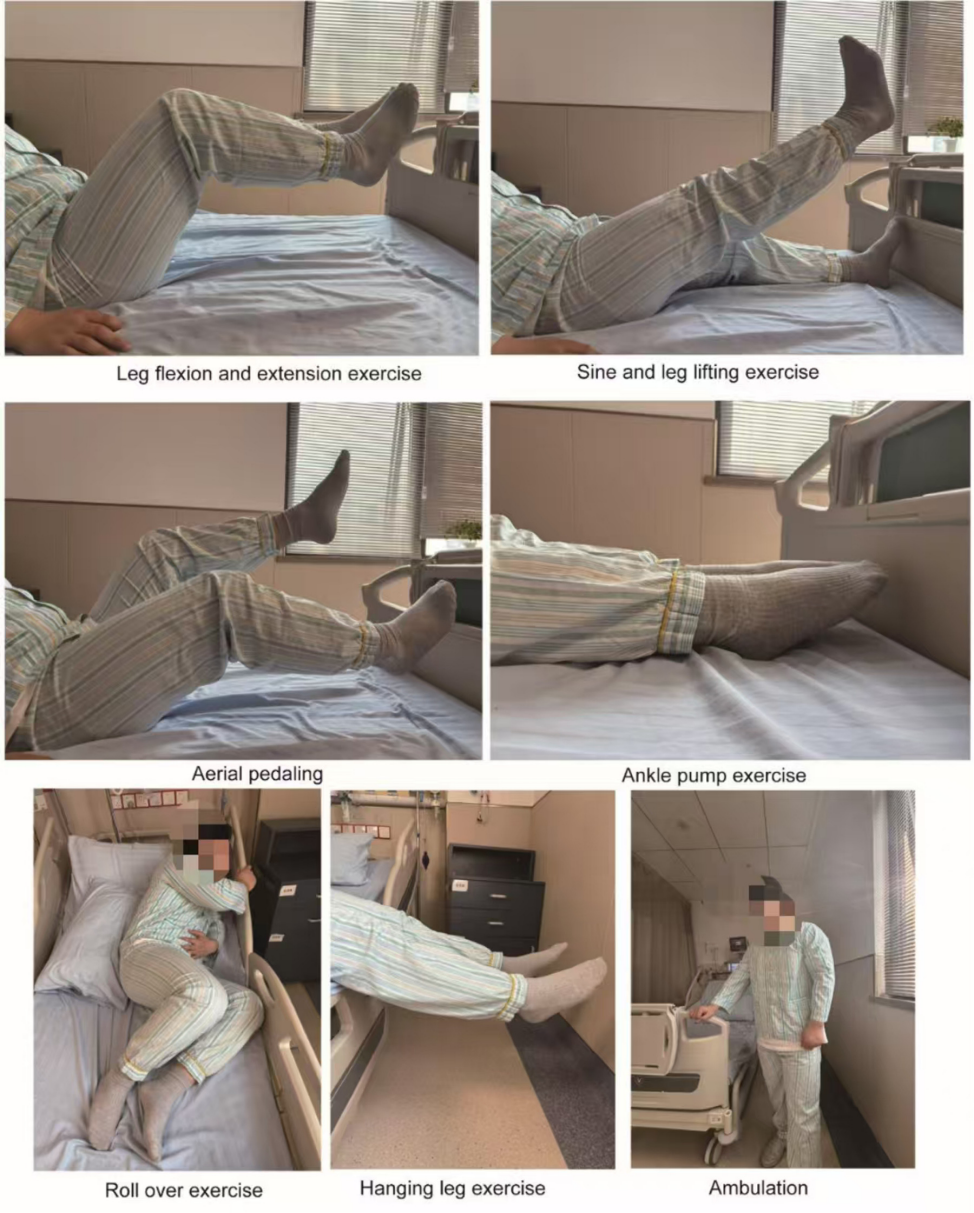
Schematic diagram of the progressive resistance exercise intervention.

While the primary focus of our study was to assess the impact of early progressive resistance exercise, other Enhanced Recovery After Surgery (ERAS) protocols were also applied to both groups. These included preoperative carbohydrate loading, reduced preoperative fasting time, and early removal of drains. However, the progressive resistance exercise was the key intervention that distinguished the two groups. The study group implemented an early progressive resistance exercise intervention protocol in addition to the routine perioperative management. A multidisciplinary intervention team was established, comprising a pancreatic surgery attending physician, a head nurse, and two nurses with over 8 years of specialized nursing experience. All team members completed systematic training in the ERAS concept and the progressive resistance exercise protocol and passed a standardized assessment to ensure the standardization and consistency of the intervention implementation. In terms of postoperative management, once the patient's vital signs were stable, the bed was adjusted to a low incline semi-recumbent position at 30° to 45° to optimize abdominal drainage and reduce abdominal wall tension. The patient's position was adjusted every 2 h. For pain management, a stepwise strategy was employed, with non-pharmacological interventions such as music therapy being prioritized. When the Visual Analog Scale (VAS) score was ≥4, non-opioid analgesics were administered according to medical orders. The nutritional support plan involved starting with small amounts of water intake (15 ml per time) 6–8 h after surgery, gradually increasing the amount of water intake within 24–48 h, and transitioning to a liquid diet after the removal of the nasogastric tube, with a gradual adjustment of the dietary structure. The exercise intervention (Figure 1) followed a phased and progressive approach. On the first postoperative day, in-bed resistance training was conducted, including elbow flexion and extension exercises using a fixed resistance band at the bedside (10 repetitions per set) and lower limb extension and bridge core training (10 repetitions per set). On the second postoperative day, the Medical Research Council (MRC) muscle strength grading was assessed, and for those who tolerated it well, bedside sitting training was added (20 min per session). On the third postoperative day, after re-assessment, those who met the criteria progressed to bedside standing (20 min per session in the morning) and short-distance walking (20 min per session in the afternoon). The interventions for both groups continued until discharge.

### 2.4 Outcome assessment

In this study, a detailed assessment of gastrointestinal function recovery and hospitalization was conducted for both groups of patients. Specific indicators included the time to first water intake, first food intake, first out-of-bed activity, and length of hospital stay. Additionally, we collected and compared the incidence of complications such as anastomotic leakage, gastrointestinal bleeding, urinary retention, and pancreatic fistula between the two groups during the intervention period. These metrics provide a direct reflection of the recovery speed of gastrointestinal function and the overall rehabilitation process post-surgery, offering crucial evidence for comparing the recovery outcomes between the two groups.

To comprehensively analyze pain, quality of life, and sleep conditions in both groups of patients, this study employed the Visual Analog Scale (VAS) (13), the European Organization for Research and Treatment of Cancer Quality of Life Questionnaire Core 30 (EORTC QLQ-C30) (14), and the Pittsburgh Sleep Quality Index (PSQI) (15). The VAS score ranges from 0–10, with higher scores indicating more severe pain; the EORTC QLQ-C30 score ranges from 0–100, with higher scores representing better quality of life (16); and the PSQI score ranges from 0 to 21, with higher scores suggesting poorer sleep quality (17). The reliability and validity of these scales have been rigorously verified. The VAS has a Cronbach's α coefficient of 0.887 and a validity of 0.858; the EORTC QLQ-C30 has a Cronbach's α coefficient of 0.824 and a validity of 0.833; and the PSQI has a Cronbach's α coefficient of 0.856 and a validity of 0.850. Assessments were performed both before and after the intervention to accurately reflect the improvement effects of the intervention on patients' pain, quality of life, and sleep.

The recovery quality in both groups of patients was assessed across five dimensions using the 40-item Quality of Recovery Score (QoR-40) (18): emotional state (9 items, 9–45 points), physical comfort (12 items, 12–60 points), psychological support (7 items, 7–35 points), self-care ability (5 items, 5–25 points), and pain (7 items, 7–35 points). The QoR-40 scale covers multiple key aspects of postoperative recovery, providing a comprehensive and systematic reflection of the patients' recovery status. Higher scores in each dimension indicate better recovery quality in that aspect (19). The scale has a Cronbach's α coefficient of 0.805 and a validity of 0.854, demonstrating good reliability and validity (20). Assessments were also conducted before and after the intervention to objectively evaluate the promoting effect of the intervention on postoperative recovery quality in patients.

### 2.5 Data analysis

Data analysis was performed using SPSS version 25.0. Categorical data were presented as frequencies and percentages (*n*/%), while continuous data were expressed as mean ± standard deviation. Comparisons between the two groups were made using the chi-square test for categorical data and the independent samples t-test for continuous data. A *p*-value of less than 0.05 was considered to indicate a statistically significant difference between the groups.

## 3 Results

**Figure 2:**
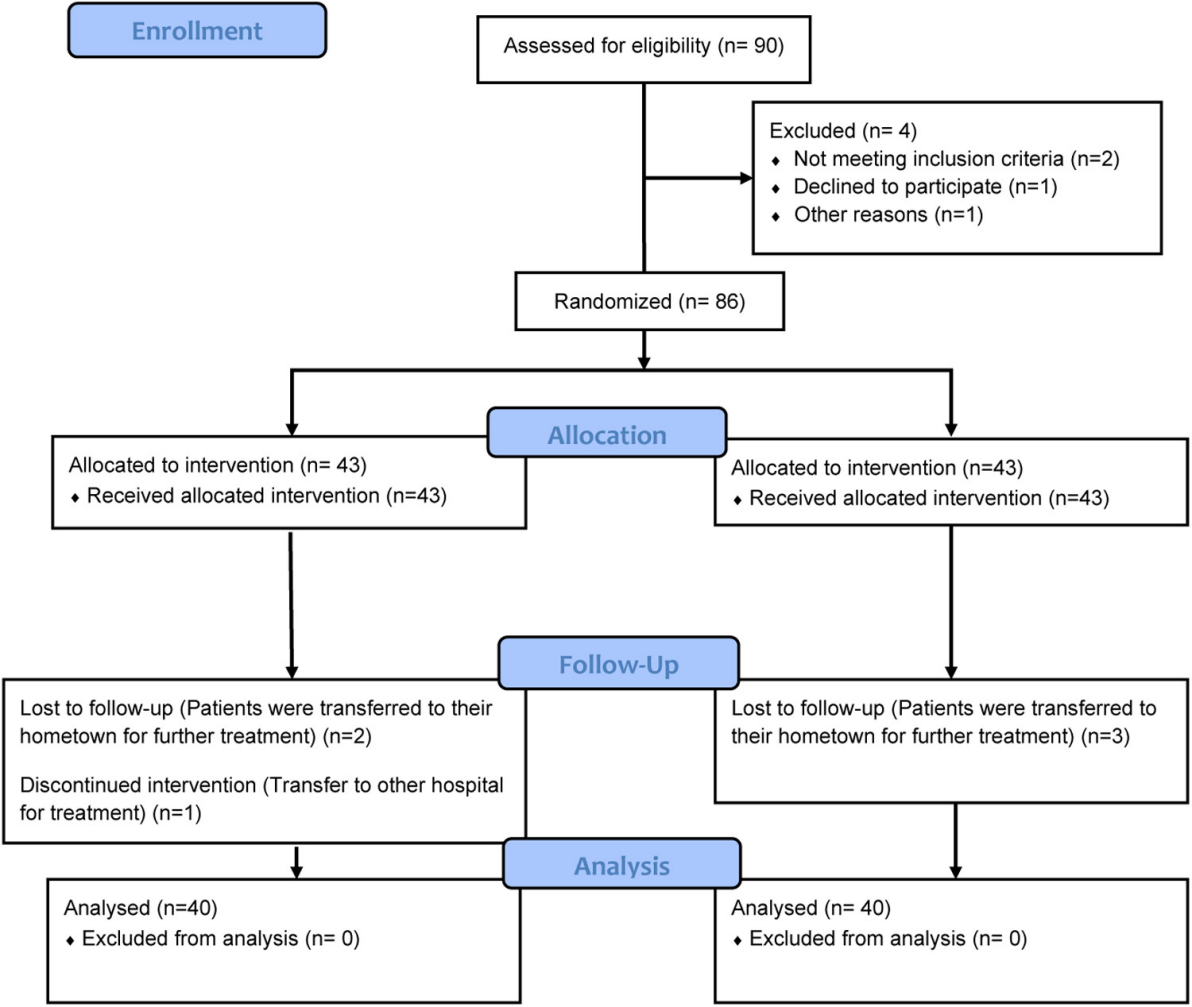
CONSORT flow diagram of patient inclusion.

As depicted in Figure 2, a total of 90 patients who met the inclusion criteria were initially identified for enrollment. Among them, 4 patients declined to participate in the study. Consequently, 86 patients were randomly assigned to either the Resistance exercise group or the Control group, with each group comprising 40 participants. During the intervention period, 5 patients withdrew from the study. Ultimately, 80 patients who underwent pancreaticoduodenectomy for pancreatic cancer were included in the final analysis, with 40 patients in each group.

As detailed in Table 1, a comprehensive comparison of baseline characteristics between the Resistance Exercise group and the Control group revealed no statistically significant differences in terms of gender distribution, mean age (years), body mass index (BMI, kg/m^2^), prevalence of comorbid conditions such as hypertension, diabetes, and hyperlipidemia, tumor diameter (cm), tumor staging, or histological classification (all *p* > 0.05). This meticulous matching of key demographic and clinical variables at the study's inception underscores the robustness of the study design and ensures that any observed differences in outcomes can be more confidently attributed to the intervention rather than preexisting disparities. Thus, the homogeneity of baseline characteristics provides a solid foundation for the subsequent comparative analyses, enhancing the internal validity and reliability of the study findings.

**Table 1:** The characteristics of patients undergoing pancreaticoduodenectomy for pancreatic cancer (*n* = 80).

Characteristic	Resistance exercise group (*n* = 40)	Control group (*n* = 40)	*t*/*χ*²	*p*
Gender			1.277	0.085
Male	23 (57.50%)	21 (52.50%)		
Female	17 (42.50%)	19 (47.50%)		
Age(y)	55.11 ± 4.48	54.85 ± 4.23	4.231	0.109
BMI (kg/m^2^)	22.02 ± 1.31	21.96 ± 1.26	2.104	0.117
Hypertension	16 (40.00%)	17 (42.50%)	1.128	0.201
Diabetes	12 (30.00%)	10 (25.00%)	1.004	0.086
Hyperlipidemia	5 (12.50%)	6 (15.00%)	1.895	0.127
Tumor diameter (cm)	3.81 ± 0.60	3.72 ± 0.59	1.382	0.098
Tumor staging			1.244	0.103
Stage Ⅰ	12(%)	13(%)		
Stage Ⅱ	19(%)	17(%)		
Stage Ⅲ	9(%)	10(%)		
Histological classification			1.821	0.079
Pancreatic ductal epithelium	29(%)	27(%)		
Non-pancreatic ductal epithelium	11(%)	13(%)		

As shown in Table 2, resistance exercise significantly shortened the time to first water intake, time to first food intake, time to first ambulation, and duration of hospital stay (all *p* < 0.05).

**Table 2:** Comparison of gastrointestinal function recovery and hospitalization outcomes between the two groups (*n* = 80).

Outcome	Resistance exercise group (*n* = 40)	Control group (*n* = 40)	t/χ²	*p*
Time to first water intake	2.36 ± 0.41	2.81 ± 0.56	1.843	0.044
Time to first food intake	5.35 ± 0.52	5.88 ± 0.55	2.037	0.019
Time to first ambulation	2.49 ± 0.52	3.23 ± 0.47	1.124	0.041
Duration of hospital stay	16.10 ± 2.51	18.83 ± 2.63	3.285	0.006

As shown in Table 3, compared with the pre-intervention status, the EORTC QLQ-C30 scores of both groups increased post-intervention, with significantly higher scores observed in the resistance exercise group (*p* < 0.05). Meanwhile, the VAS and PSQI scores of both groups decreased, with significantly lower scores in the resistance exercise group (*p* < 0.05). These findings indicate that resistance exercise is beneficial in alleviating pain, improving quality of life, and enhancing sleep quality in patients.

**Table 3:** Comparison of pain, quality of life, and sleep between the two groups before and after intervention.

Time point	Groups	VAS	EORTC QLQ-C30	PSQI
Before intervention	Resistance exercise group (*n* = 40)	5.01 ± 1.15	53.13 ± 6.85	17.01 ± 1.28
	Control group (*n* = 40)	4.87 ± 1.19	52.75 ± 6.85	16.75 ± 1.13
	*t*	1.274	8.445	4.237
	*p*	0.105	0.084	0.115
After intervention	Resistance exercise group (*n* = 40)	0.69 ± 0.36*	85.31 ± 3.24*	7.96 ± 1.64*
	Control group (*n* = 40)	1.36 ± 0.35*	68.07 ± 4.31*	12.85 ± 2.08*
	*t*	1.207	9.241	3.129
	*p*	0.035	0.020	0.003

Note: *P < 0.05 indicates a statistically significant difference compared with the pre-intervention values.

As shown in Table 4, compared with the pre-intervention status, the scores for emotional state, physical comfort, psychological support, self-care ability, and pain all increased in both groups post-intervention, with significantly higher scores observed in the resistance exercise group (*p* < 0.05). These results suggest that resistance exercise is effective in enhancing the recovery quality of patients.

**Table 4:** Comparison of recovery quality between the two groups before and after intervention.

Time point	Groups	Emotional state	Physical comfort	Psychological support	Self-care ability	Pain
Before intervention	Resistance exercise group (*n* = 40)	33.95 ± 2.27	54.15 ± 1.72	27.98 ± 1.32	11.02 ± 1.06	29.10 ± 1.24
	Control group (*n* = 40)	34.01 ± 2.11	53.86 ± 1.41	28.04 ± 1.25	10.93 ± 1.15	28.86 ± 1.22
	*t*	5.328	9.516	3.201	2.375	6.414
	*p*	0.102	0.093	0.184	0.066	0.080
After intervention	Resistance exercise group (*n* = 40)	42.42 ± 0.79*	57.26 ± 0.64*	32.45 ± 0.76*	17.14 ± 0.71*	32.10 ± 0.58*
	Control group (*n* = 40)	40.07 ± 1.26*	55.19 ± 1.16*	30.42 ± 1.17*	14.82 ± 1.25*	30.33 ± 1.23*
	*t*	8.557	8.436	4.264	3.229	5.006
	*p*	0.015	0.004	0.035	0.012	0.007

Note: *P < 0.05 indicates a statistically significant difference compared with the pre-intervention values.

As shown in Table 5, the incidence of urinary retention was significantly lower in the resistance exercise group (*p* = 0.011). There were no significant differences between the two groups in the incidence of anastomotic leak, gastrointestinal hemorrhage, and pancreatic fistula (all *p* > 0.05).

**Table 5:** Comparison of complications between the two groups.

Outcome	Resistance exercise group (*n* = 40)	Control group (*n* = 40)	χ²	*p*
Anastomotic leak	1 (2.5%)	1 (2.5%)	0.000	1.000
Gastrointestinal hemorrhage	1 (2.5%)	0 (0%)	0.988	1.000
Urinary retention	1 (2.5%)	5 (12.5%)	2.486	0.011
Pancreatic fistula	1 (2.5%)	0 (0%)	0.988	1.000

## 4 Discussion

Pancreaticoduodenectomy is one of the primary treatment options for pancreatic cancer, capable of directly resecting malignant tumor tissues to control disease progression and positively impact patient survival extension (21). However, the surgery is notably invasive, with a relatively high risk of complications, making postoperative intervention an indispensable component of comprehensive management (22). Conventional interventions encompass monitoring vital signs, medication guidance, analgesia, nutrition, maintaining drainage, and early postoperative ambulation, yet they remain relatively passive, struggling to harness patients' individual potential (23, 24). This study systematically evaluated the multidimensional impact of progressive resistance exercise on the recovery process of patients following pancreaticoduodenectomy, yielding results of significant clinical and theoretical value.

In terms of gastrointestinal function recovery, our study confirmed that progressive resistance exercise significantly shortened the time to first water intake, first food intake, and first ambulation after surgery. This finding corroborates previous research outcomes, with the underlying mechanisms likely involving multiple aspects. Firstly, progressive resistance exercise enhances the strength of abdominal and diaphragmatic muscles, thereby improving gastrointestinal motility (25). Secondly, early activity promotes abdominal blood circulation, accelerating the recovery of intestinal nerve function (26). Thirdly, exercise stimulation may modulate gastrointestinal hormone secretion via the brain-gut axis (27). Notably, the progressive resistance exercise group experienced a significantly reduced hospital stay duration, which not only alleviated patients' financial burden but also optimized the allocation of medical resources, aligning closely with the core objectives of the ERAS concept (28, 29). It has been reported that the majority of individuals undergoing abdominal surgery are either pre-frail or frail. Handgrip strength measurement, which is both simple and cost-effective, offers valuable prognostic information regarding surgical outcomes. Specifically, muscle strength assessed via handgrip dynamometry serves as a robust predictor of length of stay in surgical contexts (30).

Regarding pain management and quality of life improvement, our study achieved encouraging results. The significant advantage of the progressive resistance exercise group in EORTC QLQ-C30 scale scores suggests that this intervention not only improves physical function but also positively impacts psychosocial functioning. The substantial reduction in VAS scores may be associated with increased endorphin release induced by exercise, while regular muscle activity might reset central sensitization. Of particular significance is the improvement in PSQI scores, likely due to exercise regulating circadian rhythms and body temperature cycles, as well as alleviating postoperative anxiety (31, 32).

The assessment results regarding recovery quality revealed that progressive resistance exercise demonstrated significant advantages in dimensions such as emotional state and physical comfort. This finding supports the modern rehabilitation concept of “Exercise is Medicine” (33). Exercise may enhance cognitive function and emotional state by upregulating brain-derived neurotrophic factor (BDNF) levels while boosting patients' self-efficacy (34). In older patients with advanced cancer, adequate muscle strength is associated with longer overall survival (35). These results imply that muscle strength may be a useful indicator for estimating survival and identifying older patients who would benefit from anticancer treatment. Besides, prehabilitation programs may reduce postoperative complication rates and the frequency of emergency department visits (36).

In the context of complication prevention, the progressive resistance exercise group demonstrated a significantly lower incidence of urinary retention, a finding of considerable clinical importance (31, 37). This reduction may be attributed to several physiological mechanisms associated with the exercise intervention. Firstly, progressive resistance exercise is known to enhance the recovery of pelvic floor muscle function, which plays a crucial role in maintaining urinary continence (38). By strengthening these muscles, the exercise protocol may improve their ability to support the bladder and urethra, thereby reducing the likelihood of urinary retention. Secondly, the exercise regimen likely contributes to improved autonomic nerve regulation. The autonomic nervous system, which governs involuntary bodily functions, plays a critical role in bladder control. Enhanced regulation of the autonomic nerves through exercise can improve the coordination between the bladder's detrusor muscle and the urinary sphincter, facilitating more efficient bladder emptying (39). Additionally, the overall metabolic improvements resulting from early mobilization and increased physical activity should not be overlooked. Progressive resistance exercise promotes better circulation, oxygenation, and nutrient delivery to tissues, which can accelerate the recovery process and support the normal functioning of the urinary system (40). These combined effects of enhanced muscle function, improved autonomic regulation, and overall metabolic optimization likely contribute to the reduced incidence of urinary retention observed in the progressive resistance exercise group. Moreover, it is noteworthy that the implementation of progressive resistance exercise did not increase the risk of severe complications, such as anastomotic leak. This finding confirms the safety of incorporating such exercise into early postoperative management. The absence of adverse effects on anastomotic integrity suggests that the benefits of progressive resistance exercise may be achieved without compromising surgical outcomes, making it a viable and valuable addition to postoperative care protocols.

The innovation of this study lies in its comprehensive evaluation of the benefits of progressive resistance exercise in the recovery process following major pancreatic cancer surgery. However, several limitations exist: the relatively limited sample size, short follow-up duration, and lack of in-depth exploration of molecular mechanisms. Future research could consider expanding the sample size, extending follow-up periods, and incorporating biomarker detection to more comprehensively assess the long-term benefits and mechanisms of action of progressive resistance exercise.

## 5 Conclusions

In summary, resistance exercise significantly shortens the time to first water intake, time to first food intake, time to first ambulation, and duration of hospital stay. It also alleviates pain, improves quality of life, and enhances sleep quality in patients. Moreover, resistance exercise effectively enhances the recovery quality of patients and reduces the incidence of urinary retention. This study provides high-quality evidence-based support for the clinical application of progressive resistance exercise following pancreaticoduodenectomy. This exercise-based intervention, through its multi-target and multi-pathway mechanisms of action, significantly improves short-term patient outcomes and holds substantial potential for broader implementation. It is recommended that progressive resistance exercise be incorporated into the standard postoperative rehabilitation protocols for pancreatic cancer, with personalized exercise prescriptions tailored to individual patient conditions.

## Data Availability

The original contributions presented in the study are included in the article/Supplementary Material, further inquiries can be directed to the corresponding author.
